# Socioeconomic inequality in medication persistence in primary and secondary prevention of coronary heart disease – A population-wide electronic cohort study

**DOI:** 10.1371/journal.pone.0194081

**Published:** 2018-03-09

**Authors:** William King, Arron Lacey, James White, Daniel Farewell, Frank Dunstan, David Fone

**Affiliations:** 1 Public Health Wales, Cardiff, United Kingdom; 2 College of Medicine, Swansea University, Swansea, United Kingdom; 3 Centre for the Development and Evaluation of Complex Public Health Interventions and South East Wales Trials Unit, Cardiff University, Cardiff, United Kingdom; 4 Division of Population Medicine, Cardiff University, Cardiff, United Kingdom; University of Tampere, FINLAND

## Abstract

**Background:**

Coronary heart disease (CHD) mortality in England fell by 36% between 2000 and 2007 and it is estimated that approximately 50% of the fall was due to improved treatment uptake. Marked socio-economic inequalities in CHD mortality in the United Kingdom (UK) remain, with higher age-adjusted rates in more deprived groups. Inequalities in the persistence of medication for primary and secondary prevention of CHD may contribute to the observed social gradient and we investigated this possibility in the population of Wales (UK).

**Methods and findings:**

An electronic cohort of individuals aged over 20 (n = 1,199,342) in Wales (UK) was formed using linked data from primary and secondary care and followed for six years (2004–2010). We identified indications for medication (statins, aspirin, ACE inhibitors, clopidogrel) recommended in UK National Institute for Clinical Excellence (NICE) guidance for CHD (high risk, stable angina, stable angina plus diabetes, unstable angina, and myocardial infarction) and measured the persistence of indicated medication (time from initiation to discontinuation) across quintiles of the Welsh Index of Multiple Deprivation, an area-based measure of socio-economic inequality, using Cox regression frailty models. In models adjusted for demographic factors, CHD risk and comorbidities across 15 comparisons for persistence of the medications, none favoured the least deprived quintile, two favoured the most deprived quintile and 13 showed no significant differences.

**Conclusions:**

During our study period (2004–2010) we found no significant evidence of socio-economic inequality in the persistence of recommended medication for primary and secondary prevention of CHD.

## Introduction

Although coronary heart disease (CHD) mortality has fallen in recent decades in the UK and other high-income countries a social gradient persists, with a higher age-adjusted mortality in more socioeconomically deprived groups [1]. An IMPACT modelling study estimating the proportions of the fall in CHD mortality attributable to changes in risk factors or treatments (effectiveness and provision) found that in England and Wales, between 1981 and 2000, 58% of the fall in CHD mortality could be attributed to population-level reduction in major risk factors and 42% to treatments [2]. An IMPACT study of the period 2000–2007 in England, during which CHD mortality fell by 36%, estimated that improved uptake of treatments accounted for approximately 50% of the fall [3], with lipid-lowering therapy accounting for 14%.

There is an association between the degree of compliance with CHD-related medication and CHD outcomes that is not only due to the ‘healthy adherer’ effect–the tendency for individuals with good medication adherence to have generally healthier lifestyles [4–6]. There is also evidence that better medication compliance is associated with relative affluence [7] and that this applies to lipid-lowering therapy in CHD prevention [8–11].

A review of papers published between 1997 and 2005 that examined persistence of medication for hypertension and dyslipidaemia observed that few studies explicitly stated the definition of persistence used, and that different measures of persistence were employed [12]. A meta-analysis of papers published between 2000 and 2005 examining compliance with medication for hypertension, diabetes and dyslipidaemia reported that different measures of persistence used over different time frames yielded widely differing results [4]. Studies of medication compliance have used definitions and terminology differently (compliance sometimes regarded as synonymous with adherence, and sometimes as including both adherence and persistence) and therefore standard definitions are proposed [12,13], clarifying that adherence is the proportion of prescribed doses taken in the prescribed time interval and persistence is the accumulation of time from the initiation of therapy to discontinuation of therapy.

Although health care in the UK, including Wales, is publicly funded and free at the point of delivery, the concept of the ‘inverse care law’ [14] (more deprived groups having greater health care needs yet receiving poorer health care) has been influential in public health medicine in the UK. Evidence from some UK studies suggested that more socio-economically deprived groups received poorer health care for cardiovascular disease [15,16]. Our previous study of the overall pathway of CHD health care in the population of Wales [17] identified indications for recommended interventions, examined whether these interventions were received, and measured time to their delivery. The result showed little evidence of inequity (inequality to the disadvantage of more deprived groups) other than in relation to revascularization procedures. The analyses did not examine whether recommended interventions continued to be received appropriately and therefore did not exclude the possibility of inequity in medication persistence. We investigated that possibility in the same cohort of over one million individuals during the study period, 2004–2010, measuring the accumulation of time from initiation to discontinuation of recommended medication (persistence). As in our previous study [17] the recommendations for medication were those of national guidelines in primary and secondary prevention of CHD, and we used area-based measures of socio-economic deprivation. Our data enabled us to identify indications for medication in individuals, to specify the start date of each indication, and to use a time-to-event survival technique directly measuring the accumulation of time from initiation to discontinuation of therapy. We were able to adjust for relevant covariates and the data used (with the exception of the inference of deprivation from area level measures) was available at the individual level. We are not aware of any other study of this size addressing this issue in any UK population.

## Methods

The analyses were carried out within the Secure Anonymized Information Linkage databank (SAIL) at Swansea University [18,19]. The system allows researchers to link primary care data, hospital activity data, and mortality data to inform the clinical history of individuals.

Permission to undertake the analyses was obtained from the Information Governance Review Panel at SAIL in line with the Collaborative Review System (project reference number 0156).

### Datasets

We defined a retrospective cohort of individuals aged 20 or over, resident in Wales and registered with SAIL-submitting general practices between 1 January 2004 and 31 December 2010. Individuals reaching the age of 20 were added to the cohort at annual time points. Routine data from the Welsh Demographic Service, Patient Episode Data for Wales (PEDW) hospital admission data (ICD 10 and Office of Population and Censuses codes for CHD-related hospital episodes and procedures), and primary care data (Read codes for diagnosis, investigation and treatment of CHD and the prescribing of antihypertensive, lipid-lowering and anti-platelet therapy) were extracted to form a linked dataset. If an individual died or ceased to be registered with a SAIL-submitting practice this was treated as a censoring event. The primary care data available for our study was available only from SAIL-submitting practices, covering approximately 40% of the population of Wales. There is no available evidence that these practices were unrepresentative. The distribution of urban and rural residency of the population resembled that of Wales as a whole. Comparison with Office of National Statistics (ONS) mid-year data (2004) for the whole of Wales showed small differences in age distribution, our cohort having 1.2% fewer in the proportion aged over 40. Comparison of the socio-economic distribution of our study population with that of Wales as a whole showed that quintiles 1 and 3 were over-represented (21.3% and 22.94% respectively) and quintiles 2, 4 and 5 were under-represented (18.34%, 18.72% and 18.55% respectively).

### Assessment of socioeconomic inequalities

The Welsh Index of Multiple Deprivation (WIMD) (2008) for the individual’s residence was assessed at the Lower Super Output Area (LSOA) (mean population 1500) as a measure of socioeconomic deprivation. The 2008 WIMD is based on the resident’s income, employment status, education, housing, health and geographical access to services [20].

Inequalities were examined by comparing WIMD quintile 5 (most deprived) to quintile 1 (least deprived).

### Indicated medication

The criteria for indicated medication were based on the interventions recommended in UK National Service Frameworks (NSFs) for CHD [21,22] and National Institute for Health and Care Excellence (NICE) guidelines relating to the study period [23–27]. In relation to primary prevention, the level of CHD event risk at which statin treatment was recommended by NSF and NICE guidelines was 20% over ten years [27]. Components of the risk score were systolic blood pressure, body mass index (BMI), smoking status, and cholesterol: high density lipoprotein (HDL) ratio. Those whose risk of CHD was designated high on this basis are referred to as ‘risk assessed high’ (see Table 1) and those at high risk because of a diagnosis of cerebrovascular disease, peripheral vascular disease or diabetes are referred to as ‘high-risk diagnosis’.

**Table 1:** Numbers of medications initiated for each indication and discontinued within observation period by indication and medication type.

Original indication	Medication initiated	Number initiating medication	Number stopping medication
Risk assessed high	Statin	33228	5378
High-risk diagnosis	Statin	29208	4041
Stable angina	Statin	11231	1711
Stable angina and diabetes	Statin	6588	782
Unstable angina	Statin	9211	1341
MI	Statin	11380	1642
Stable angina	Aspirin	10704	2590
Stable angina and diabetes	Aspirin	5472	1056
Unstable angina	Aspirin	8663	1654
MI	Aspirin	11011	1889
Stable angina and diabetes	ACE inhibitor	5620	827
Unstable angina	ACE inhibitor	7860	1443
MI	ACE inhibitor	10772	1916
Unstable angina	Clopidogrel	5783	3419
MI	Clopidogrel	10133	6536

We developed a set of algorithms based on Read codes [28] to identify all individuals for whom medication in primary and secondary prevention of CHD was indicated, and we examined provision and persistence of that medication. The 15 interventions for which we made comparisons between deprivation groups that are shown in Table 1. We did not make comparisons for the numerous possible combinations of medications used in management of hypertension.

Our routine data did not provide information on individuals’ contraindications to medication, or refusal of treatment. Data were not available on in-patient prescribing and our examination of prescription of medication was confined to the period after individuals left hospital.

Although the recommended interval between of repeat prescriptions in UK general practice is usually 28 days there is variation in practice, and there is evidence that in Wales during our study period a minority of repeat prescriptions were issued at intervals longer than longer 28 days [29, 30]. We made a simplifying assumption and designated the point at which persistence of medication had lapsed as the last issue of that prescription where it occurred at least 56 days prior to the end of the observation period for that individual.

### Covariates

We examined a number of potentially confounding factors determined at the time-point at which the indication for the medication appeared. They were selected on the basis of availability and evidence from previous studies [17]. Covariates available included demographic factors (age, sex); risk-factors (systolic blood pressure, body mass index, smoking status, cholesterol: HDL ratio); co-morbidities based on the Charlson co-morbidity index [31] collapsed to a binary variable because some components were already considered as covariates (CHD, cerebrovascular disease and diabetes); and the Framingham non-laboratory risk assessment score (comprising sex, age, systolic blood pressure, BMI, smoking status, reported diabetic status, and current treatment for hypertension) [32]. We accounted for previous indications for the same medication: for example statins indicated by angina might previously have been indicated by risk-assessment.

### Statistical methods

We used a Cox model with random effects (frailty models) to examine the persistence of CHD-related medication, measuring the length of time from initiation until the issue of the last prescription for medication that appeared still to be indicated (with at least 56 days without a prescription prior to the end of the observation period required to define a last prescription). For each of the comparisons we produced Kaplan-Meier plots that enabled treatment of censoring events.

We fitted the individual’s general practice or admitting hospital modelled as random effects, to allow for unobserved hospital or primary care specific factors that might cause clustering. The type 1 error probability was set to 0.05 for all analyses. Modelling was performed using the *coxme* package in R [33].

We used multiple imputations (n = 20 imputed datasets) with chained equations to impute values for missing covariates: systolic blood pressure, BMI, cholesterol:HDL ratio, smoking status and admission type, including all outcomes in the prediction model. We performed sensitivity analyses, re-running analyses using the 2001 Townsend deprivation quintiles and the 1991 Framingham assessment tool.

## Results

The initial cohort comprised 1,201,399 subjects. After exclusion of those with an incorrectly coded date of birth (n = 202), absent coding for gender (n = 7) or with discontinuous registration with SAIL (n = 1848) the cohort was reduced to 1,199,342.

**Fig 1:**
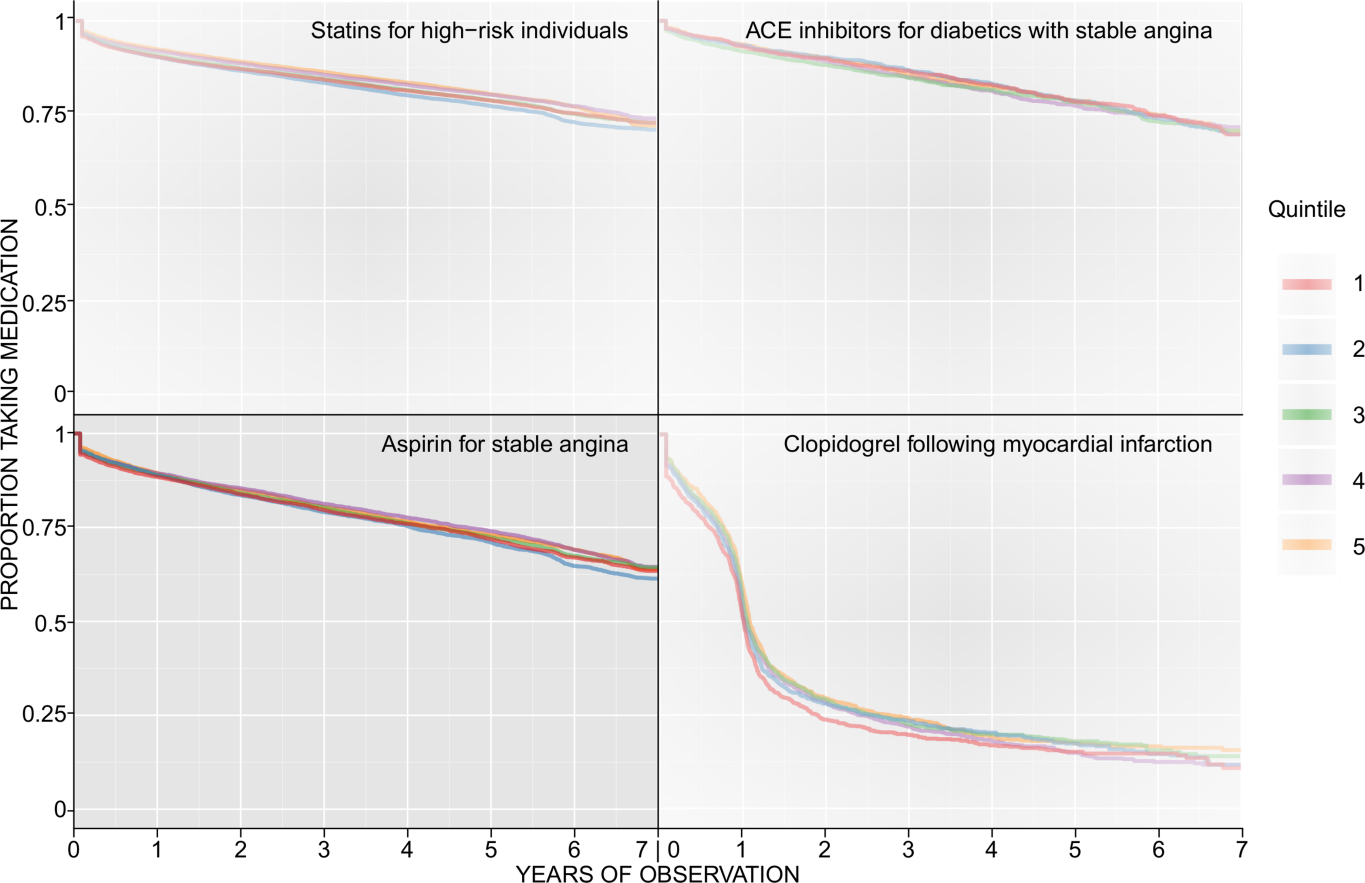
Panel of Kaplan-Meier plots for selected indications and medications showing the discontinuation in medication persistence across the seven year observation period. Within each individual Kaplan-Meier plot, the survival curve is shown separately for each of the deprivation quintiles. Quintile 1 (red) is the least deprived quintile.

Table 1 shows the number of medications initiated for each indication and the number of those treatments subsequently discontinued during the observation period. The pattern of discontinuation is shown in the panel of Kaplan-Meier plots (Fig 1).

One plot for each of the medications investigated is shown. The patterns shown for each of the drugs for these indications are reflected in very similar patterns for each drug for the other indications investigated. Additional Kaplan-Meier plots for these indications are included in the supporting information for this paper; those shown are illustrative of the patterns observed.

For statins, ACE inhibitors and aspirin, a gradual decrease in persistence was observed over the seven years of observation. At five years, 75% of individuals initiated on statins for ‘risk assessed high’ were still being prescribed the medication in each of the deprivation quintiles. A similar pattern was observed for ACE inhibitors and aspirin, although the proportion of individuals still prescribed these medications at five years was slightly less than 75% for each of the quintiles. For clopidogrel, the recommendation that applied during the period of our study [23] for 12 months of treatment is reflected in the pronounced decline in persistence at one year following initiation of therapy, with a gradual decline thereafter. Each of the plots shows a decline in persistence following the initial 28-day prescription, with this pattern most pronounced for clopidogrel. Importantly, no substantial differences in persistence are observed between deprivation quintiles.

A summary of our results in the fully adjusted models is presented in Table 2. Two comparisons showed statistically significant differences: in individuals with a ‘high-risk diagnosis’ the persistence for statins favoured the most deprived quintile (HR 0.78, 95% CI 0.70, 0.88), and in individuals who had a myocardial infarction (MI) the persistence for clopidogrel (during the 12-month period for which it was indicated post-MI) also favoured the most deprived quintile (HR 0.86, 95% CI 0.78, 0.95).

**Table 2:** Hazard ratios (HRs) and 95% confidence intervals (CIs) for the association between socioeconomic inequalities and discontinuation of indicated medication for CHD prevention. Hazard ratios (HRs) relate to discontinuation of medication during the indicated period. Hazard ratios less than 1 indicate that the most deprived quintile was less likely than the least deprived quintile to stop taking the medication for the given indication. HR adjusted for age, sex, smoking status, BMI, hypertension, cholesterol: HDL ratio, and comorbidities; for MI and unstable angina indications also adjusted for admission type and specialty.

Original indication	Medication initiated	Hazard ratio for comparison between quintile 5 and quintile 1 (95% confidence interval)	Statistically significant difference?	Favours
Risk assessed high	Statin	0.95 (0.86, 1.05)	No	
High-risk diagnosis	Statin	0.78 (0.70, 0.88)	Yes	Most deprived
Stable angina	Statin	1.06 (0.88, 1.26)	No	
Stable angina and diabetes	Statin	0.84 (0.65, 1.09)	No	
Unstable angina	Statin	1.04 (0.84, 1.27)	No	
MI	Statin	0.97 (0.80, 1.18)	No	
Stable angina	Aspirin	1.01 (0.88, 1.17)	No	
Stable angina and diabetes	Aspirin	0.86 (0.69, 1.08)	No	
Unstable angina	Aspirin	0.85 (0.71, 1.02)	No	
MI	Aspirin	1.13 (0.94, 1.35)	No	
Stable angina and diabetes	ACE inhibitor	1.10 (0.85, 1.41)	No	
Unstable angina	ACE inhibitor	1.14 (0.93, 1.38)	No	
MI	ACE inhibitor	1.15 (0.96, 1.36)	No	
Unstable angina	Clopidogrel	1.03 (0.91, 1.17)	No	
MI	Clopidogrel	0.86 (0.78, 0.95)	Yes	Most deprived

Use of the Framingham 1991 risk assessment tool and the Townsend deprivation quintiles in ours sensitivity analyses made only small differences to the estimated hazard ratios and of the 15 comparisons the same two (for ‘high risk diagnosis’ and for MI) significantly favoured quintile 5. For ‘high risk diagnosis’ the hazard ratio was 0.78 in the fully adjusted model, 0.79 in the Framingham 1991 risk assessment analysis and 0.82 in the Townsend quintiles analysis. For MI the hazard ratios in these analyses were respectively 0.86, 0.86 and 0.88. In none of the models was there a significant difference in any of the 13 other comparisons and therefore the overall pattern remained unchanged in the sensitivity analyses.

## Discussion

We found little evidence of socio-economic inequality in persistence of indicated medication in the primary and secondary prevention of CHD. Only two of the 15 indicated medications showed a difference in persistence according to levels of socio-economic deprivation, both having a longer persistence in the most deprived groups.

Persistence of medication depends on healthcare-related factors, including administrative systems, the observing and recording of indications for medication, and individual prescription review [34, 35]. The introduction in the UK in 2004 of the Quality and Outcomes Framework (QOF), a major public health intervention, incentivised such activities [36]. Persistence also depends on patient-factors including adverse reactions, emerging contra-indications, refusal of treatment, and comorbidities. Our findings indicate that the overall effects of these factors (healthcare-related and patient-related) did not cause inequity in persistence of the medication we examined.

### Comparison with other studies

Our findings are consistent with those from a UK study on the use of statins after first myocardial infarction during the period 1997–2004 [8] that found approximately 80% of individuals continued to receive statins at one year and approximately 76% at 5–10 year follow-up.

In a multinational, multicentre trial of individuals with acute coronary syndromes (ACS), 1999–2003, discontinuation rates at six months were 8% for aspirin, 13% for statins, 20% for ACE inhibitors [37]. In a USA study individuals undergoing catheterization for CHD between 1998–2001 were found to have discontinuation rates at 12 months of 28% for ACE inhibitors and 18% for aspirin [38]. In a UK study of individuals after MI (2003–2009) the adjusted odds of being prescribed clopidogrel at 12 months were 53% for NSTEMI and 54% for STEMI [39].

A number of large (> 30, 000 subjects) UK studies, ecological or population-based, have examined evidence of equity in use of CHD-related medication although none primarily examined persistence. Of the seven large studies using prescribing rates to examine lipid-lowering therapy in primary prevention of CHD the results in four favoured the less deprived [40–44], two showed no difference [15, 16] and one favoured the more deprived group [45]. Of eight large UK studies examining use of anti-platelet medication (aspirin and clopidogrel) in primary and/or secondary prevention of CHD the results in primary prevention in two studies favoured the less deprived [15, 16], in one favoured the more deprived [46], and in two showed no difference [40,47]. In relation to anti-platelet medication in secondary prevention the results in one study favoured the less deprived [15], in two favoured the more deprived [48,49], and in one showed no significant difference [50]. Four studies indicating inequity in lipid-lowering therapy in primary prevention were published between 1998 and 2004, [40–43], but a large individual-level study examining the increase in utilization of medication, but not examining persistence, found no evidence of inequity in secondary prevention of CHD in the UK between 1999 and 2007 [49]. Our study found that in both primary and secondary prevention of CHD between 2004 and 2011 there was no significant evidence of inequity in medication persistence.

### Policy implications

Our study period coincided with a relatively rapid improvement in the UK in a range of clinical activity indicators, including those related to the recording and delivery of CHD-related primary care [51]. This was attributed partly to the introduction in 2000 of the National Service Framework for CHD in England (2001 in Wales) and in 2004 of the Quality Outcomes Framework (QOF) [36]. Our findings suggest that if these improvements extended to the appropriate persistence of medication in CHD healthcare they did so equitably.

### Strengths of the study

We based our study on data from a large number of individuals (more than one million) and used a time-to-event methodology developed to address population-level analysis of healthcare use adjusted for need.

Our study examined a health service in which healthcare is free at the point of delivery, and in Wales, unlike other parts of the UK, there is no charge for prescriptions. It has been shown in the USA that a lower level of insurance cover, and consequently higher financial cost to the individual, is associated with poorer adherence to medication used in secondary prevention of CHD [52]. The integrated system of healthcare in the UK differs significantly from systems such as those seen in the USA and results from the study of our population may form a useful comparator for studies in other healthcare systems.

### Limitations of the study

Because of the complexity of recommended medication for hypertension with numerous potential permutations, and because of the difficult in distinguishing their clinical indications in an individual (as when a diuretic or a beta blocker might have been prescribed for reasons other than hypertension) we did not include antihypertensives in this analysis, although we analysed the use of ACE inhibitors in relation to stable angina with diabetes, unstable angina, and MI. Area-based measures of socio-economic status are known to underestimate inequalities and their use may therefore have reduced our ability to detect inequalities.

### Conclusions

We found no significant evidence of inequity in medication persistence in primary and secondary prevention of CHD.

## Abbreviations

LSOA: lower super output area
NICE: National Institute for Clinical Excellence (1999–2005), National Institute for Health and Clinical Evidence (2005–2013), National Institute for Health and Care Excellence (2013-)
NSF: National Service Framework
ONS: Office of National Statistics
PEDW: Patient Episode Data for Wales
QOF: Quality Outcomes Framework
SAIL: Secure Anonymised Information Linkage
WIMD: Welsh Index of Multiple Deprivation

## References

[1] McCartney D, Scarborough P, Webster P, Rayner M. Trends in social inequalities for premature coronary heart disease mortality in Great Britain, 1994–2008: a time trend ecological study. BMJ Open. 2012 1 1;2(3):e000737 doi: 10.1136/bmjopen-2011-000737 22710128 10.1136/bmjopen-2011-000737 PMC3378944

[2] Unal B, Critchley JA, Capewell S. Explaining the decline in coronary heart disease mortality in England and Wales between 1981 and 2000. Circulation. 2004 3 9;109(9):1101–7. doi: 10.1161/01.CIR.0000118498.35499.B2 14993137 10.1161/01.CIR.0000118498.35499.B2

[3] Bajekal M, Scholes S, Love H, Hawkins N, O'Flaherty M, Raine R, et al Analysing recent socioeconomic trends in coronary heart disease mortality in England, 2000–2007: a population modelling study. PLoS medicine. 2012 6 12;9(6):e1001237 doi: 10.1371/journal.pmed.1001237 22719232 10.1371/journal.pmed.1001237 PMC3373639

[4] Cramer JA, Benedict A, Muszbek N, Keskinaslan A, Khan ZM. The significance of compliance and persistence in the treatment of diabetes, hypertension and dyslipidaemia: a review. Int J Clin Pract. 2008 1 1;62(1):76–87. doi: 10.1111/j.1742-1241.2007.01630.x 17983433 10.1111/j.1742-1241.2007.01630.x PMC2228386

[5] Ho PM, Magid DJ, Shetterly SM, Olson KL, Maddox TM, Peterson PN, et al Medication nonadherence is associated with a broad range of adverse outcomes in patients with coronary artery disease. Am Heart J. 2008 4 30;155(4):772–9. doi: 10.1016/j.ahj.2007.12.011 18371492 10.1016/j.ahj.2007.12.011

[6] Brookhart MA, Patrick AR, Dormuth C, Avorn J, Shrank W, Cadarette SM, et al Adherence to lipid-lowering therapy and the use of preventive health services: an investigation of the healthy user effect. Am J Epidemiol. 2007 8 1;166(3):348–54. doi: 10.1093/aje/kwm070 17504779 10.1093/aje/kwm070

[7] Capewell S, Graham H. Will cardiovascular disease prevention widen health inequalities? PLoS Med. 2010 8 24;7(8):e1000320 doi: 10.1371/journal.pmed.1000320 20811492 10.1371/journal.pmed.1000320 PMC2927551

[8] Carey IM, DeWilde S, Shah SM, Harris T, Whincup PH, Cook DG. Statin use after first myocardial infarction in UK men and women from 1997 to 2006: Who started and who continued treatment? Nutr Metab Cardiovasc Dis. 2012 5 31;22(5):400–8. doi: 10.1016/j.numecd.2010.09.010 21194912 10.1016/j.numecd.2010.09.010

[9] Mann DM, Allegrante JP, Natarajan S, Halm EA, Charlson M. Predictors of adherence to statins for primary prevention. Cardiovasc Drugs Ther. 2007 8 1;21(4):311–6. doi: 10.1007/s10557-007-6040-4 17665294 10.1007/s10557-007-6040-4

[10] Bates TR, Connaughton VM, Watts GF. Non-adherence to statin therapy: a major challenge for preventive cardiology. Expert Opin Pharmacother. 2009 12 1;10(18):2973–85. doi: 10.1517/14656560903376186 19954271 10.1517/14656560903376186

[11] Liberopoulos EN, Florentin M, Mikhailidis DP, Elisaf MS. Compliance with lipid-lowering therapy and its impact on cardiovascular morbidity and mortality. Expert Opin Drug Saf. 2008 11 1;7(6):717–25. doi: 10.1517/14740330802396984 18983218 10.1517/14740330802396984

[12] Caetano PA, Lam JM, Morgan SG. Toward a standard definition and measurement of persistence with drug therapy: examples from research on statin and antihypertensive utilization. Clin Ther. 2006; 28: 1411–1424. doi: 10.1016/j.clinthera.2006.09.021 17062314 10.1016/j.clinthera.2006.09.021

[13] Cramer JA, Roy A, Burrell A, Fairchild CJ, Fuldeore MJ, Ollendorf DA, et al Medication compliance and persistence: terminology and definitions. Value Health. 2008; 11: 44–47. doi: 10.1111/j.1524-4733.2007.00213.x 18237359 10.1111/j.1524-4733.2007.00213.x

[14] Hart JT. The inverse care law. The Lancet. 1971 2 27;297 (7696):405–12. 10.1016/s0140-6736(71)92410-x 4100731

[15] Saxena S, Car J, Eldred D, Soljak M, Majeed A.; Practice size, caseload, deprivation and quality of care of patients with coronary heart disease, hypertension and stroke in primary care: national cross sectional study; BMC Health Serv Res 2007 6 27;7(1). 10.1186/1472-6963-7-96 PMC1919365 17597518

[16] Ward PR, Noyce PR, St Leger AS. Exploring the equity of GP practice prescribing rates for selected coronary heart disease drugs: a multiple regression analysis with proxies of healthcare need. Int J Equity Health. 2005 2 8;4(1):3 doi: 10.1186/1475-9276-4-3 15701165 10.1186/1475-9276-4-3 PMC548940

[17] King W, Lacey A, White J, Farewell D, Dunstan F, Fone D. Equity in healthcare for coronary heart disease, Wales (UK) 2004–2010: A population-based electronic cohort study. PloS one. 2017 3 16;12(3):e0172618 doi: 10.1371/journal.pone.0172618 28301496 10.1371/journal.pone.0172618 PMC5354260

[18] Ford DV, Jones KH, Verplancke JP, Lyons RA, John G, Brown G, et al The SAIL Databank: building a national architecture for e-health research and evaluation. BMC Health Serv Res. 2009 9 4;9(1):157. 19732426 10.1186/1472-6963-9-157 PMC2744675

[19] Lyons RA, Jones KH, John G, Brooks CJ, Verplanke J-P, Ford DV, et al The SAIL databank: linking multiple health and social care datasets. BMC Med Inform DecisionMaking. 2009;9(3) 10.1186/1472-6947-9-3 PMC2648953 19149883

[20] Welsh index of multiple deprivation 2008: Technical report; http://wales.gov.uk/topics/statistics/publications/publication-archive/wimd2008tech/?lang=en;2009

[21] Department of Health. National Service Framework for Coronary Heart Disease. 2000 (Department of Health, London).

[22] National Service Framework for Coronary Heart Disease. 2001. (National Assembly for Wales, Cardiff)

[23] National Institute for Clinical Excellence (2001) Prophylaxis for patients who have experienced a myocardial infarction. (CGA)

[24] National Institute for Clinical Excellence (2002) Management of type 2 diabetes–management of blood pressure and blood lipids (CGH)

[25] National Institute for Health and Clinical Excellence (2004) Hypertension: management of hypertension in adults in primary care (CG18)

[26] National Institute for Health and Clinical Excellence (2006) Hypertension: management of hypertension in adults in primary care (CG34)

[27] National Institute for Health and Clinical Excellence (2008) Lipid modification: Cardiovascular risk assessment and the modification of blood lipids for the primary and secondary prevention of cardiovascular disease (CG67) 25340243

[28] Bentley T, Price C, Brown P; Structural and lexical features of successive versions on the Read codes; in The Proceedings of the 1996 Annual Conference of The Primary Health Care Specialist Group; 1996.

[29] All Wales Medicines Strategy Group. All Wales Review and Guidance for Prescribing Intervals. 2013. Available at: http://www.awmsg.org/docs/awmsg/medman/All%20Wales%20Review%20and%20Guidance%20for%20Prescribing%20Intervals.pdf. Accessed December 2016

[30] O’Dowd A. Some PCTs recommend GPs limit prescriptions to 28 days. BMJ 2011;342:d2410

[31] Charlson ME, Pompei P, Ales KL, MacKenzie CR. A new method of classifying prognostic comorbidity in longitudinal studies: development and validation. J Chronic Dis. 1987 12 31;40(5):373–83. 3558716 10.1016/0021-9681(87)90171-8

[32] D'Agostino RB Sr, Vasan RS, Pencina MJ, Wolf PA, Cobain M, Massaro JM, et al (2008) General cardiovascular risk profile for use in primary care: the Framingham Heart Study. Circulation 117: 743–753. doi: 10.1161/CIRCULATIONAHA.107.699579 18212285 10.1161/CIRCULATIONAHA.107.699579

[33] Therneau T. coxme: Mixed effects Cox models. R package version. 2012;2(3).

[34] Avery AJ, Sheikh A, Hurwitz B, Smeaton L, Chen YF, Howard R, et al Safer medicines management in primary care. Br J Gen Pract. 2002 10 1;52(Suppl):S17–22. 12389765 PMC1316136

[35] Harris CM, Dajda R. The scale of repeat prescribing. Br J Gen Pract. 1996 11 1;46(412):649–53. 8978110 PMC1239818

[36] Doran T, Fullwood C, Gravelle H, Reeves D, Kontopantelis E, Hiroeh U, et al Pay-for-performance programs in family practices in the United Kingdom. N Engl J Med. 2006 7 27;355(4):375–84. doi: 10.1056/NEJMsa055505 16870916 10.1056/NEJMsa055505

[37] Eagle KA, Kline-Rogers E, Goodman SG, Gurfinkel EP, Avezum A, Flather MD, et al Adherence to evidence-based therapies after discharge for acute coronary syndromes: an ongoing prospective, observational study. Am J Med. 2004 7 15;117(2):73–81. doi: 10.1016/j.amjmed.2003.12.041 15234641 10.1016/j.amjmed.2003.12.041

[38] Kulkarni SP, Alexander KP, Lytle B, Heiss G, Peterson ED. Long-term adherence with cardiovascular drug regimens. Am Heart J. 2006 1 31;151(1):185–91. doi: 10.1016/j.ahj.2005.02.038 16368315 10.1016/j.ahj.2005.02.038

[39] Boggon R, van Staa TP, Timmis A, Hemingway H, Ray KK, Begg A, et al Clopidogrel discontinuation after acute coronary syndromes: frequency, predictors and associations with death and myocardial infarction—a hospital registry-primary care linked cohort (MINAP–GPRD). Eur Heart J. 2011 8 29;32(19):2376–86. doi: 10.1093/eurheartj/ehr340 21875855 10.1093/eurheartj/ehr340 PMC3184230

[40] Ward PR, Noyce PR, St Leger AS. Are GP practice prescribing rates for coronary heart disease drugs equitable? A cross sectional analysis in four primary care trusts in England. J Epidemiol Community Health. 2004 2 1;58(2):89–96. doi: 10.1136/jech.58.2.89 14729882 10.1136/jech.58.2.89 PMC1732682

[41] Packham C, Pearson J, Robinson J, Gray D. Use of statins in general practices, 1996–8: cross sectional study. BMJ. 2000 6 10;320(7249):1583–4. 10845969 10.1136/bmj.320.7249.1583 PMC27405

[42] Packham C, Robinson J, Morris J, Richards C, Marks P, Gray D. Statin prescribing in Nottingham general practices: a cross-sectional study. J Public Health (Oxf). 1999 3 1;21(1):60–4. 10.1093/pubmed/21.1.60 10321861

[43] Bradshaw NS, Fone DL, Walker R. Equity of health care: a ward-based analysis of primary care prescribing. Pharm J.1998;261:R11-.

[44] Ward P R, Noyce P R, St Leger A S; How equitable are GP practice prescribing rates for statins?: an ecological study in four primary care trusts in North West England. Int J Equity Health 2007 3 27;6(1): 2. 17386118 10.1186/1475-9276-6-2 PMC1847516

[45] Ashworth M, Lloyd D, Smith R S, Wagner A, Rowlands G. Social deprivation and statin prescribing: a cross-sectional analysis using data from the new UK general practitioner ‘Quality and Outcomes Framework’; J Public Health (Oxf) 2007 3 1;29(1): 40–7. 17071815 10.1093/pubmed/fdl068

[46] Vinogradova Y, Leighton M, Avery A, Logan RF, Kendrick D, Atherton JC, et al; 408 Increased aspirin use and upper gastrointestinal bleeding rates in socially deprived patients; Gastroenterology 2009 5 1;136(5): A–69

[47] Petty D, Silcock J; Explanations for variations in clopidogrel prescribing in England; J Public Health (Oxf) 2008 12 1;30(4):494–8. 18591212 10.1093/pubmed/fdn048

[48] McGovern M P, Williams D J, Hannaford P C, Taylor MW, Lefevre KE, Boroujerdi MA et al; Introduction of a new incentive and target-based contract for family physicians in the UK: good for older patients with diabetes but less good for women?. Diabetic Medicine. 2008 9 1;25(9):1083–9. doi: 10.1111/j.1464-5491.2008.02544.x 18937676 10.1111/j.1464-5491.2008.02544.x

[49] Hawkins N, Scholes S, Bajekal M, Love H, O’Flaherty M, Raine R, et al; The UK National Health Service. Circulation: Cardiovascular Quality and Outcomes. 2013 1 1:CIRCOUTCOMES-111. 10.1161/CIRCOUTCOMES.111.000058 23481523

[50] McLean G, Sutton M, Guthrie B; Deprivation and quality of primary care services: evidence for persistence of the inverse care law from the UK Quality and Outcomes framework. J Epidemiol Community Health. 2006 11 1;60(11):917–22. doi: 10.1136/jech.2005.044628 17053278 10.1136/jech.2005.044628 PMC2465488

[51] Doran T, Fullwood C, Kontopantelis E, Reeves D. Effect of financial incentives on inequalities in the delivery of primary clinical care in England: analysis of clinical activity indicators for the quality and outcomes framework. The Lancet. 2008 9 5;372(9640):728–36. 10.1016/S0140-6736(08)61123-X 18701159

[52] Choudhry NK, Avorn J, Glynn RJ, Antman EM, Schneeweiss S, Toscano M, et al Full coverage for preventive medications after myocardial infarction. N Eng J Med. 2011 12 1;365(22):2088–97. 10.1056/NEJMsa1107913 22080794

